# Key scientific issues in developing drinking water guidelines for perfluoroalkyl acids: Contaminants of emerging concern

**DOI:** 10.1371/journal.pbio.2002855

**Published:** 2017-12-20

**Authors:** Gloria B. Post, Jessie A. Gleason, Keith R. Cooper

**Affiliations:** 1 New Jersey Department of Environmental Protection, Trenton, New Jersey, United States of America; 2 New Jersey Department of Health, Trenton, New Jersey, United States of America; 3 Rutgers University, New Brunswick, New Jersey, United States of America; National Institute of Environmental Health Sciences, United States of America

## Abstract

Perfluoroalkyl acids (PFAAs), a group of synthetic organic chemicals with industrial and commercial uses, are of current concern because of increasing awareness of their presence in drinking water and their potential to cause adverse health effects. PFAAs are distinctive among persistent, bioaccumulative, and toxic (PBT) contaminants because they are water soluble and do not break down in the environment. This commentary discusses scientific and risk assessment issues that impact the development of drinking water guidelines for PFAAs, including choice of toxicological endpoints, uncertainty factors, and exposure assumptions used as their basis. In experimental animals, PFAAs cause toxicity to the liver, the immune, endocrine, and male reproductive systems, and the developing fetus and neonate. Low-dose effects include persistent delays in mammary gland development (perfluorooctanoic acid; PFOA) and suppression of immune response (perfluorooctane sulfonate; PFOS). In humans, even general population level exposures to some PFAAs are associated with health effects such as increased serum lipids and liver enzymes, decreased vaccine response, and decreased birth weight. Ongoing exposures to even relatively low drinking water concentrations of long-chain PFAAs substantially increase human body burdens, which remain elevated for many years after exposure ends. Notably, infants are a sensitive subpopulation for PFAA’s developmental effects and receive higher exposures than adults from the same drinking water source. This information, as well as emerging data from future studies, should be considered in the development of health-protective and scientifically sound guidelines for PFAAs in drinking water.

This Perspective is part of the *Challenges in Environmental Health: Closing the Gap between Evidence and Regulations Collection*.

## Introduction

Perfluoroalkyl acids (PFAAs), a group of persistent organic pollutants, are the focus of current attention because of their detection in drinking water and a rapid increase in evidence for their adverse health effects. This commentary discusses PFAAs as emerging drinking water contaminants, and key scientific data and risk assessment issues for development of health-protective and scientifically sound PFAA drinking water guidelines.

**Fig 1:**
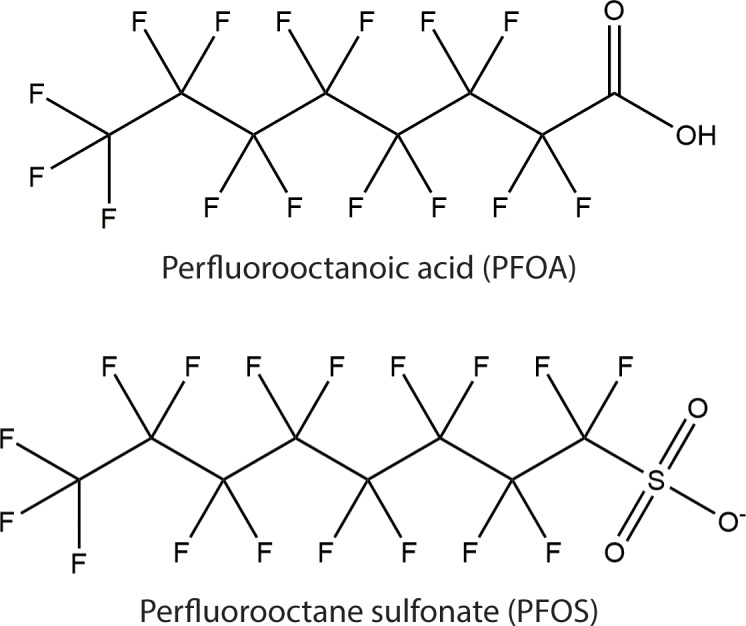
Structures of PFOA and PFOS.

PFAAs (often referred to by the broader term, perfluorinated chemicals [PFCs]) are anthropogenic compounds with a totally fluorinated carbon chain and a negatively charged functional group such as carboxylate or sulfonate (Fig 1). They are part of the larger group, per- and polyfluoroalkyl substances (PFAS), that encompasses many other aliphatic substances containing at least one totally fluorinated carbon atom [1]. Although the most well-known and well-studied PFAAs are the eight-carbon compounds, perfluorooctanoic acid (PFOA) and perfluorooctane sulfonate (PFOS), PFAAs with longer or shorter carbon chains as well as other types of PFAS also occur in drinking water and are also the subject of current research [2–4].

PFAAs have been produced for over 60 years. They are used commercially and in industrial processes because they repel both oil and water, withstand elevated temperatures, and are highly resistant to chemical reactions. Commercial applications include stain-resistant coatings for upholstery and carpeting, water-resistant breathable outdoor clothing, and greaseproof food packaging. They are used in the manufacture of fluoropolymers such as polytetrafluoroethylene in non-stick cookware and fluoroelastomers (high-performance synthetic rubber), but are not intentionally present in these finished products [1,5]. PFAAs and numerous other PFAS are also found in aqueous film-forming foams (AFFF) used to fight hydrocarbon fires [6,7].

## Why are PFAAs in drinking water a current concern?

PFAAs differ from other environmental contaminants in several important ways. They persist indefinitely in the environment because of the strength of their carbon-fluorine bonds. PFAAs are highly water-soluble while other well-known persistent, bioaccumulative, and toxic (PBT) organic pollutants such as polychlorinated dioxins and polychlorinated biphenyls have low water solubility. In contrast to PFAAs, drinking water is not a major exposure source for these other PBT contaminants [8].

Sources of PFAAs in the environment include industrial facilities where they are made or used, release of AFFF during training or firefighting, industrial and domestic wastewater treatment plant effluent, land application of biosolids (sludge), and leachate from industrial waste or consumer products disposed of in landfills [8]. Major United States manufacturers have voluntarily ended production and use of long-chain PFAAs (i.e., perfluorinated carboxylates with eight or more carbons and perfluorinated sulfonates with six or more carbons) and their precursors, due to concerns about biological persistence and potential health effects [5,9]. However, environmental contamination is expected to continue due to their environmental persistence, continued formation from precursors, and ongoing production by nonparticipating manufacturers especially overseas, particularly in China and India [5,10]. Of particular note, PFAAs that reach groundwater may remain there indefinitely, impacting drinking water sources for generations to come [11].

While not the focus of this paper, it is important to mention current concerns about the numerous compounds that have been introduced as replacements for the phased-out long-chain PFAAs, including shorter chain PFAAs and other types of PFAS such as perfluoroethers [12]. While these replacements are generally less bioaccumulative than long-chain PFAAs, they are similarly environmentally persistent, may be more mobile in the environment, and are less efficiently removed from drinking water by standard treatment processes [13,14]. They are not detected by standard analytical methods but have been found in drinking water at levels of potential concern in research studies [15,16]. Furthermore, some replacements cause toxic effects similar to long-chain PFAAs [17], while the toxicity of others may not have been adequately studied [13].

Both the scientific literature and awareness of PFAAs in US drinking water have greatly increased in the past decade. A PubMed search for the keyword “PFOA” and synonyms located only 244 citations before 2006, 667 from 2006–2010, and over 1,100 from 2011–2015. Similarly, PFAA occurrence was investigated in only a few US public water systems (PWS) near sites of industrial release until New Jersey conducted the first statewide studies of PFAAs in drinking water, which included 54 PWS, in 2006 and 2009–2010 [3,18].

The recent US Environmental Protection Agency (EPA) Unregulated Contaminant Monitoring Rule 3 (UCMR3) study revealed previously unknown PFAA contamination in PWS throughout the US [4]. UCMR3 required testing of all large US PWS (serving >10,000 people) and a limited sample of smaller PWS for six PFAAs from 2013–2015. PFAAs were reported in 194 US PWS (about 4% of those tested) serving about 16.5 million people in 36 states and territories [19]. AFFF, particularly from discharge at military bases, is identified as the source of many PFAA detections in UCMR3 including in some PWS with the highest levels.

UCMR3 reporting thresholds were much higher (e.g., 20 ng/L for PFOA; 40 ng/L for PFOS) than in other studies of PFAAs in PWS such as the two conducted in New Jersey (4–5 ng/L; [3,18]). Because of these differences in reporting thresholds, PFAAs were detected several times more frequently in the two New Jersey studies than in New Jersey UCMR3 monitoring. Similarly, PFAAs are also present at levels too low to be reported in UCMR3 in many additional PWS in other states [20]. Although PFAAs from industrial sources or AFFF were recently discovered in several small US PWS, most small PWS were not tested in UCMR3. Additionally, PFAAs have been detected in private drinking water wells adjacent to sources (e.g., [21,22]), but many such wells remain untested.

Long-chain PFAAs including PFOA, PFOS, perfluorononanoic acid (PFNA; the nine-carbon carboxylate), and perfluorohexane sulfonate (PFHxS; the six-carbon sulfonate) are found in the low parts per billion (ng/ml) range in the blood serum of almost all residents of the US and many other nations [23,24]. Body burdens of these compounds result from exposures to both the compounds themselves and conversion of precursors in the body [25]. These long-chain PFAAs are not metabolized and are slowly excreted with human half-lives of several years. Thus, PFAA serum levels remain elevated for many years after exposure ends. In contrast, short-chain PFAAs such as the four-carbon compounds perfluorobutanoic acid (PFBA) and perfluorobutane sulfonate (PFBS) are eliminated much faster, with half-lives of 3 and 26 days, respectively [26,27].

**Fig 2:**
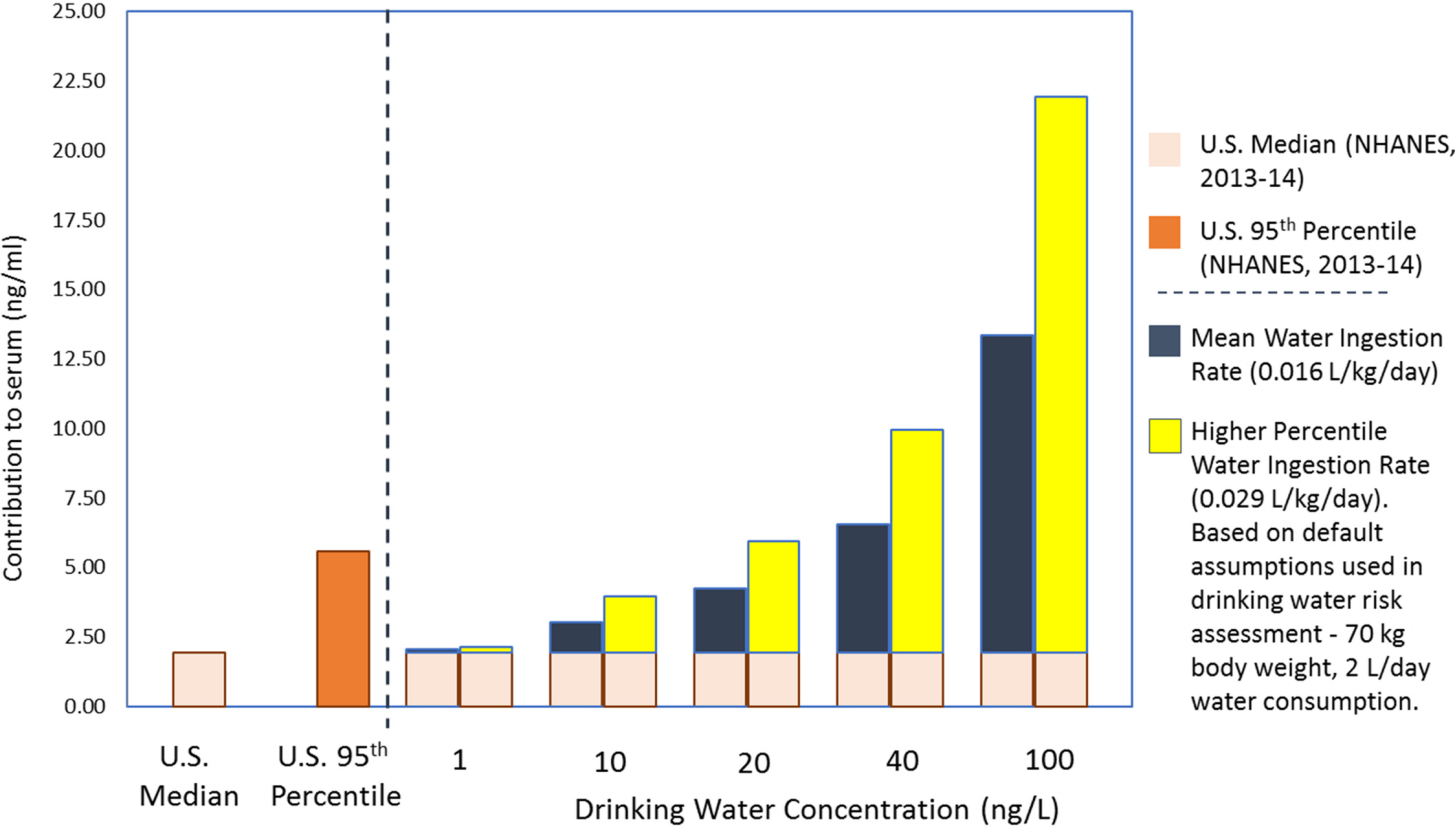
Predicted increases in serum PFOA concentrations from consumption of drinking water with various concentrations of PFOA. Predicted serum PFOA concentrations from consumption at mean [30] and upper percentile drinking water ingestion rates, as compared to median and 95th serum PFOA concentration percentiles from NHANES [23]. Predictions are based on the clearance factor for PFOA (0.14 ml/kg/day), which relates PFOA dose (ng/kg/day) to serum PFOA concentration (ng/ml) [31–33]. PFOA, perfluorooctanoic acid; NHANES, National Health and Nutrition Examination Survey.

While general population exposure to PFAAs and precursors comes from sources including diet and consumer products, studies of exposed communities and predictions based on toxicokinetic factors show that low levels of PFAAs in drinking water (i.e., well below 100 ng/L [parts per trillion]) substantially increase blood serum levels. These empirical observations and toxicokinetic models (Fig 2) consistently demonstrate that serum PFOA levels in adults increase on average by more than 100 times the drinking water concentration [8,28], with greater predicted increases for PFOS and PFNA. Notably, serum PFOA and PFOS were significantly higher among individuals residing in zip codes with UCMR3 detections than in other zip codes, although the study design tended to minimize differences between these two groups [29].

## Scientific information considered in developing PFAA drinking water guidelines

Health-based guidelines for drinking water contaminants are developed with risk assessment approaches that consider toxicological, epidemiological, and mode of action data, and they are most often primarily based on toxicological data from laboratory animals. PFOA and PFOS have more health effects information than most other contaminants with drinking water guidelines, and toxicological data are sufficient for guideline development for other PFAAs such as PFNA, PFBA, and PFBS. However, for some PFAAs of concern in drinking water, particularly PFHxS, additional toxicological studies are likely needed before guidelines can be developed.

Numerous human studies have examined associations between PFAAs and many health endpoints. For example, over 130 such studies of PFOS were published through 2015. A strength of most of these studies is that associations are based on blood serum PFAA levels that reflect individual variations in both external exposure (e.g., amount of water ingested each day) and physiological parameters affecting excretion rates [31]. Exposure assessments based on serum PFAA levels are therefore less uncertain than external exposure measures such as residential drinking water concentrations.

Human studies come from the general population, such as the Centers for Disease Control’s National Health and Nutrition Examination Survey (NHANES), a nationally representative sample of the US population [34,35]. For PFOA, a wealth of data for a wide range of health endpoints comes from the C8 Health Study of approximately 70,000 Ohio and West Virginia residents exposed for at least 1 year to drinking water concentrations of 50 ng/L to >3,000 ng/L [36]. However, such large-scale studies have not been conducted in communities whose drinking water is contaminated with PFOS, other long-chain PFAAs, or the complex PFAS mixtures found in AFFF. Finally, health effects of several PFAAs were studied in occupationally exposed workers [34].

For PFOA, the PFAA with the largest epidemiological database, associations are generally consistent for increases in cholesterol, certain liver enzymes, and uric acid in blood serum; decreased fetal growth; and decreased vaccine response [31]. Testicular and kidney cancer were also among several outcomes linked to PFOA in drinking water in the C8 Health Study. For other effects, no associations were found, or the data may be too limited to make firm conclusions [31].

A distinctive feature of the dose-response curves for several effects (e.g., increased serum lipids and liver enzymes) is that they are steepest at low exposures, including those prevalent in the general population, with a much flatter slope approaching a plateau at higher exposures. A potential explanation for the absence of associations in some occupational studies, although associations are found in less exposed populations, is that the exposures of even the least exposed workers who serve as controls may be high enough to fall on the “plateau” portion of the dose-response curve [8,31]. Similarly, associations may not be observed in studies in which all subjects are from communities with drinking water exposures sufficient to reach the “plateau” [31].

Although some effects linked to PFOA are relatively small in magnitude, they present a public health concern because such population-level changes can shift the overall distribution, thereby increasing the number of individuals with clinically abnormal values. Additionally, small changes in a clinical measure such as birth weight may be indicative of other effects that were not evaluated such as changes in subtler developmental parameters [31,37].

Laboratory animal studies identify multiple targets for PFAA toxicity including the liver, immune system, endocrine system, male reproductive system, and developing fetus and neonate [31,38,39]. Toxicological effects are generally concordant with human epidemiology data, and recent studies suggest that dietary factors (high-fat Western human diet versus low-fat standard laboratory rodent diet) may contribute to differences in effects on lipid metabolism observed in rodents versus humans [40,41]. In chronic rodent carcinogenicity studies, PFOA and PFOS caused tumors while perfluorohexanoic acid (PFHxA), a six-carbon carboxylate which is more rapidly excreted, did not [42]. Toxicity that persists into adulthood from low-dose prenatal or neonatal exposures to some PFAAs is of particular concern. For PFOA, such effects, including changes in mammary gland development and persistent liver toxicity, are the most sensitive known toxicological endpoints [31].

Understanding the mode of action is crucial to the risk assessment process used to develop health-based guidelines, particularly in evaluating whether laboratory animal data are relevant to humans. An important mode of action for PFAAs is activation of cellular receptors that regulate expression of genes controlling many biological pathways [43]. Activation can occur from even very low concentrations of compounds with high specificity for the receptor.

A longstanding question about human relevance of PFAAs’ effects in rodents involves the role of the nuclear receptor, peroxisome proliferator-activated receptor-α (PPAR-α). PPAR-α is found in many tissues and has important roles in physiological processes including energy homeostasis, lipid metabolism, inflammation, and reproduction and development [44–47]. Most PFAAs activate PPAR-α to some degree, and certain toxic effects of some PFAAs occur wholly or partially via PPAR-α while other effects are PPAR-α independent [43–48].

PPAR-α is functional in humans, as shown by the efficacy of PPAR-α activators such as fibrates in decreasing serum lipids. However, the human relevance of rodent liver tumors that occur through PPAR-α activation is subject to debate because levels and/or intrinsic activity of hepatic PPAR-α are lower in humans [49]. Unfortunately, this uncertainty about liver tumors has been the basis for generalizations about PFAAs that are not scientifically supportable, including dismissing human relevance of all rodent hepatic effects, all PPAR-α mediated non-hepatic (immune and developmental) rodent effects, or even all rodent data [50].

Data from multiple sources including monkeys, standard rodent strains, genetically modified mice lacking PPAR-α or expressing human PPAR-α, and in vitro receptor activation studies demonstrate that these generalizations are not valid. Hepatic effects of PFOS are clearly PPAR-α independent, and both PPAR-α dependent and independent processes are involved in hepatic effects of PFOA and PFNA [8,31,37,39]. Furthermore, humans are not known to be less susceptible than rodents to PPAR-α–mediated effects on the immune system or development, and some important effects in rodents such as neonatal mortality and low-dose immune suppression caused by PFOS are PPAR-α independent [37,51,52].

## Key risk assessment issues in development of PFAA guidelines

Most recent health-based drinking water guidelines for long-chain PFAAs developed by EPA and several states in the US range from 13–70 ng/L, far lower than earlier guidelines based on older scientific literature (Table 1). Drinking water guidelines for PFAAs differ based on the choice of toxicological endpoint, uncertainty factors, drinking water exposure assumptions (e.g., for average adult, lactating woman, or infant), and assumed exposure from non-drinking water sources. Some academic and other scientists suggest that guidelines as low as ≤ 1 ng/L are needed based on epidemiological findings for PFOA and PFOS, particularly decreased vaccine response, and low-dose developmental effects of PFOA such as delayed mammary gland development in mice [53]. Although detailed discussion of the basis of the various guidelines is beyond the scope of this commentary, several important issues in development of drinking water guidelines for PFAAs should be mentioned.

**Table 1:** EPA and state health-based drinking water guidelines for long-chain PFAAs^a^.

PFAA	Source	Year	Guideline (ng/L)
PFOA	EPA [33]	2016	70^b^
	Minnesota [54]	2017	35
	New Jersey [31]	2017	14^c^
	North Carolina [55]	2006	2,000
	Texas [56]	2016	290
	Vermont [57]	2016	20^b^
PFOS	EPA [33]	2016	70^b^
	Minnesota [54]	2017	27
	Texas [56]	2016	560
	Vermont [57]	2016	20^b^
PFNA	New Jersey [39]	2015	13^c^
	Texas [56]	2016	290
PFHxS	Texas [56]	2016	93

**Abbreviations:** EPA, US Environmental Protection Agency; PFHxS, perfluorohexane sulfonate; PFNA, perfluorononanoic acid; PFOA, perfluorooctanoic acid; PFOS, perfluorooctane sulfonate.

^a^ Includes drinking water guidelines and ground water guidelines applied to public water systems and/or private drinking water wells. Guidelines of several additional states that are not listed are based on EPA drinking water guidelines [33].

^b^ Applies to total of PFOA and PFOS.

^c^ Drinking water standards recommended by New Jersey Drinking Water Quality Institute. For PFNA, New Jersey groundwater standard of 10 ng/L has same basis.

Human studies are preferred as the basis for drinking water guidelines when suitable data are available. However, there is a high bar for use of human epidemiology in quantitative risk assessment due to its observational nature. Human data support a causal relationship between exposure to some PFAAs, particularly PFOA, and certain health effects (e.g., increases in serum lipids and certain liver enzymes). However, limitations in the current human database, such as inability to determine the dose-response relationships for individual PFAAs due to co-occurrence of other PFAAs, preclude the use of human data as the primary basis for PFAA drinking water guidelines. Accordingly, animal data are the primary basis for all PFAA drinking water guidelines developed so far by governmental organizations. This approach should be reconsidered if future studies provide further support for use of human data.

Although current guidelines are not based on human data, considerable evidence linking some PFAAs with multiple human health effects even within the general population exposure range indicates the need for caution about additional exposure from drinking water [31]. Therefore, health-protective guidelines for PFAAs must consider the resulting increase in blood serum levels (Fig 2). Unfortunately, this has not always been the case; for example, the EPA PFOA guideline of 70 ng/L [58] will increase average blood serum levels from the general population median of about 2 parts per billion (ppb) to about 10 ppb, with much greater increases in infants [31]. Links with several health effects at lower serum levels indicate that increases of this magnitude are not desirable and may not be protective of public health.

An important consideration in risk assessments is the choice of the toxicological endpoint(s) used as their basis. Drinking water guidelines for PFAAs are generally based on non-cancer effects, and guidelines based on sensitive non-cancer PFOA endpoints, such as the value recommended by the New Jersey Drinking Water Quality Institute [31], protect for carcinogenicity at the one-in-one-million lifetime risk level.

For PFOA, hepatic effects, delayed bone formation, and accelerated puberty are the primary basis for recent guidelines [31,58], while more sensitive developmental endpoints such as delayed mammary gland development would result in much lower values [8,31,59,60]. Delayed mammary gland development from low doses of PFOA is well established, and several studies linking PFOA with shorter duration of breastfeeding support potential human relevance [31]. However, this effect has not been used as the primary basis for PFOA risk assessment for reasons including lack of precedent, although it and other low-dose effects are accounted for in some PFOA guidelines with an uncertainty factor used when “there is concern that future studies may identify a more sensitive effect, target organ, population, or lifestage” [31]. If additional studies provide further support for this or other emerging toxicological endpoints, their use as the primary basis for PFOA risk assessment should be reconsidered.

Similarly, the recent EPA (2016) drinking water guideline for PFOS is based on decreased offspring body weight. However, review of the toxicological literature suggests consideration of decreased immune response as a more sensitive and appropriate basis [60], and Minnesota’s recent PFOS guideline adds an additional uncertainty factor to EPA’s assessment to account for potential immunotoxicity at lower doses [61].

Because PFAA half-lives are much longer in humans than animals, serum levels are much greater in humans than animals given the same dose. The default uncertainty factor of 3 for interspecies toxicokinetic variability is insufficient to account for this large difference. Therefore, comparison of animals and humans is based on internal doses, as indicated by blood serum levels, in most current drinking water guidelines for long-chain PFAAs (e.g., [31,39,58]).

Serum PFAA levels in breastfed infants are typically several fold higher than in older individuals using the same contaminated drinking water source; infants consuming formula prepared with contaminated water also receive higher exposures than adults (reviewed in [31,39]). This is of concern because developmental effects from early life exposures are sensitive endpoints for PFAA toxicity. These higher exposures are accounted for in Vermont’s guidelines by basing exposure on an infant ingestion rate that is larger than the default adult drinking water ingestion rate [57]. However, this approach is uncertain because PFAA risk assessments are based on steady-state serum levels from constant doses over many years, while infant exposures vary with age and occur over a period too short to reach steady-state. This problem has recently been addressed by Minnesota’s development of guidelines based on toxicokinetic models that predict infant PFAA exposures from breast milk and formula [61,62].

Another consideration in developing PFAA drinking water guidelines is the Relative Source Contribution (RSC) factor, which accounts for assumed exposure from non-drinking water (e.g., food, consumer products) [63]. The default RSC is 20%; i.e., it is assumed that 20% of total exposure comes from drinking water and 80% from other sources. However, a chemical-specific RSC between 20% and 80%, which results in a less stringent guideline, may be derived when supported by available data. Most drinking water guidelines for PFAAs use the default RSC because it is the most public health-protective option and because non-drinking water exposures in communities with drinking water contamination are not fully characterized (e.g., [31,33]). However, some guidelines use higher chemical-specific RSCs based on the assumption that 95th percentile general population serum PFAA concentrations represent an upper limit for non-drinking water exposures [39,61,62].

Finally, multiple PFAAs with potentially additive or synergistic toxicities often co-occur in drinking water. Because the dose-response for some effects is steepest at low exposures and approaches a plateau at higher exposures, dose-response for mixtures may be complex and dose-dependent. Although cumulative effects are not considered in most PFAA drinking water guidelines, the EPA guideline of 70 ng/L and the Vermont guideline of 20 ng/L apply to PFOA and PFOS individually, as well as the sum of both [58].

PFAAs that have drinking water guidelines may also co-occur with other PFAS not included in routine analysis that are detected only by research analytical methods [15]. Such nontarget analytes may include many components of the complex PFAS mixtures found in AFFF, as well as PFAS such as the perfluoroethers used as industrial replacements for phased-out long-chain PFAAs [7,13,15]. An important but frequently overlooked benefit of addressing exceedances of PFAA drinking water guidelines is that treatment removal processes intended to remove the compound(s) of concern may also partially or totally remove other PFAAs and PFAS, as well as unrelated contaminants, that may be present at levels of public health concern [31].

In conclusion, long-chain PFAAs cause low-dose toxicological effects in animals and some are associated with human health effects at general population exposure levels. Ongoing exposure to even relatively low drinking water concentrations of long-chain PFAAs substantially increases human body burdens, which remain elevated for many years after exposure ends. Additionally, infants, a sensitive subpopulation, receive much higher exposures than adults from the same drinking water source. This information, along with other considerations presented above and additional data from future studies, should be considered in the development of PFAA drinking water guidelines to ensure that they are health-protective and scientifically sound.

## Abbreviations

AFFF: aqueous film-forming foams
EPA: US Environmental Protection Agency
NHANES: National Health and Nutrition Examination Survey
PBT: persistent, bioaccumulative, and toxic
PFAA: perfluoroalkyl acid
PFAS: per- and polyfluoroalkyl substances
PFBA: perfluorobutanoic acid
PFBS: perfluorobutane sulfonate
PFC: perfluorinated chemical
PFHxA: perfluorohexanoic acid
PFHxS: perfluorohexane sulfonate
PFNA: perfluorononanoic acid
PFOA: perfluorooctanoic acid
PFOS: perfluorooctane sulfonate
PPAR-α: peroxisome proliferator-activated receptor-α
ppb: parts per billion
PWS: public water system
RSC: Relative Source Contribution
UCMR3: Unregulated Contaminant Monitoring Rule 3
